# Results From an Italian Expanded Access Program on Cannabidiol Treatment in Highly Refractory Dravet Syndrome and Lennox–Gastaut Syndrome

**DOI:** 10.3389/fneur.2021.673135

**Published:** 2021-05-20

**Authors:** Luigi Francesco Iannone, Gabriele Arena, Domenica Battaglia, Francesca Bisulli, Paolo Bonanni, Antonella Boni, Maria Paola Canevini, Gaetano Cantalupo, Elisabetta Cesaroni, Manuela Contin, Antonietta Coppola, Duccio Maria Cordelli, Giovanni Cricchiuti, Valentina De Giorgis, Maria Fulvia De Leva, Marta De Rinaldis, Giuseppe d'Orsi, Maurizio Elia, Carlo Andrea Galimberti, Alessandra Morano, Tiziana Granata, Renzo Guerrini, Monica A. M. Lodi, Angela La Neve, Francesca Marchese, Silvia Masnada, Roberto Michelucci, Margherita Nosadini, Nicola Pilolli, Dario Pruna, Francesca Ragona, Anna Rosati, Margherita Santucci, Alberto Spalice, Nicola Pietrafusa, Pasquale Striano, Elena Tartara, Laura Tassi, Amanda Papa, Claudio Zucca, Emilio Russo, Oriano Mecarelli, The CBD LICE Italy Study Group

**Affiliations:** ^1^Science of Health Department, School of Medicine, University Magna Graecia, Catanzaro, Italy; ^2^Pediatric Neurology, Department of Woman and Child Health and Public Health, Child Health Area, A. Gemelli University Polyclinic Foundation, Istituti di Ricovero e Cura a Carattere Scientifico (IRCCS), Catholic University of the Sacred Heart, Rome, Italy; ^3^Department of Biomedical and Neuromotor Sciences, University of Bologna, Bologna, Italy; ^4^Epilepsy Center (Reference Center for Rare and Complex Epilepsies - EpiCARE), Istituti di Ricovero e Cura a Carattere Scientifico (IRCCS) Istituto delle Scienze Neurologiche di Bologna, Bologna, Italy; ^5^Epilepsy and Clinical Neurophysiology Unit, Istituti di Ricovero e Cura a Carattere Scientifico (IRCCS) Eugenio Medea, Scientific Institute, Treviso, Italy; ^6^Child Neuropsichiatry, Istituti di Ricovero e Cura a Carattere Scientifico (IRCCS) Istituto Delle Scienze Neurologiche di Bologna, Bologna, Italy; ^7^Department of Health Sciences, Epilepsy Center, San Paolo Hospital, University of Milan, Milan, Italy; ^8^Child Neuropsychiatry, Department of Surgical Sciences, Dentistry, Gynecology, and Pediatrics, University of Verona, Verona, Italy; ^9^Child Neurology and Psychiatry Unit, G. Salesi Children's Hospital-University of Ancona, Ancona, Italy; ^10^Istituti di Ricovero e Cura a Carattere Scientifico (IRCCS) Istituto Delle Scienze Neurologiche di Bologna, Bologna, Italy; ^11^Department of Neuroscience, Reproductive, and Odontostomatological Sciences, Epilepsy Centre, University of Naples Federico II, Naples, Italy; ^12^IRCCS Istituto delle Scienze Neurologiche di Bologna, UOC Neuropsichiatria dell'età Pediatrica, Bologna, Italy; ^13^Department of Pediatrics, Epilepsy Center, Institute of Medicine, University Hospital of Udine, Udine, Italy; ^14^Department of Child Neurology and Psychiatry, Istituti di Ricovero e Cura a Carattere Scientifico (IRCCS) Mondino Foundation, Pavia, Italy; ^15^Pediatric Neurology, Department of Neuroscience, Santobono-Pausilipon Children's Hospital, Naples, Italy; ^16^Unit for Severe Disabilities in Developmental Age and Young Adults (Developmental Neurology and Neurorehabilitation), Scientific Institute Istituti di Ricovero e Cura a Carattere Scientifico (IRCCS) “E. Medea”, Brindisi, Italy; ^17^Epilepsy Centre - Clinic of Nervous System Diseases, Riuniti Hospital, Foggia, Italy; ^18^Oasi Research Institute Istituti di Ricovero e Cura a Carattere Scientifico (IRCCS), Troina, Italy; ^19^Epilepsy Center, Istituti di Ricovero e Cura a Carattere Scientifico (IRCCS) Mondino Foundation, Pavia, Italy; ^20^Neurology Unit, Department of Human Neurosciences, “Sapienza” University, Rome, Italy; ^21^Department of Pediatric Neuroscience, Fondazione Istituti di Ricovero e Cura a Carattere Scientifico (IRCCS) Istituto Neurologico Carlo Besta, Milan, Italy; ^22^Pediatric Neurology, Neurogenetics, and Neurobiology Unit and Laboratories, A. Meyer Children's Hospital, Florence, Italy; ^23^Pediatric Neurology Unit, Epilepsy Center, Department of Neuroscience, “Fatebenefratelli e Oftalmico” Hospital, Milan, Italy; ^24^Department of Basic Medical Sciences, Neurosciences and Sense Organs, University of Bari, Bari, Italy; ^25^Pediatric Neurology and Muscular Diseases Unit, Istituti di Ricovero e Cura a Carattere Scientifico (IRCCS) 'G. Gaslini' Institute, Genoa, Italy; ^26^Department of Neurosciences, Rehabilitation, Ophthalmology, Genetics, Maternal, and Child Health, University of Genoa, Genoa, Italy; ^27^Department of Pediatric Neurology, V. Buzzi Children's Hospital, Milan, Italy; ^28^Istituti di Ricovero e Cura a Carattere Scientifico (IRCCS) Istituto delle Scienze Neurologiche di Bologna, Unit of Neurology, Bellaria Hospital, Bologna, Italy; ^29^Paediatric Neurology and Neurophysiology Unit, Department of Women's and Children's Health, University Hospital of Padua, Padua, Italy; ^30^Pediatric Neurology and Epileptology Unit, Brotzu Hospital Trust, Cagliari, Italy; ^31^Child Neurology Division, Department of Pediatrics, Sapienza University of Rome, Rome, Italy; ^32^Neurology Unit, Department of Neurosciences (N.S., M.T.), Bambino Gesù Children's Hospital, Istituti di Ricovero e Cura a Carattere Scientifico (IRCCS), Rome, Italy; ^33^Claudio Munari” Epilepsy Surgery Centre, Azienda Socio Sanitaria Territoriale (ASST) Grande Ospedale Metropolitano Niguarda, Milan, Italy; ^34^Child Neuropsychiatry Department, Maggiore della Carità University Hospital, Novara, Italy; ^35^Clinical Neurophysiology Unit, Istituti di Ricovero e Cura a Carattere Scientifico (IRCCS) Eugenio Medea, Scientific Institute, Lecco, Italy; ^36^Department of Human Neurosciences, Sapienza University, Rome, Italy

**Keywords:** cannabidiol, epilepsy, Dravet syndrome, lennox-gastaut syndrome, expanded access program

## Abstract

**Background:** Purified cannabidiol (CBD) was administered to highly refractory patients with Dravet (DS) or Lennox–Gastaut (LGS) syndromes in an ongoing expanded access program (EAP). Herein, we report interim results on CBD safety and seizure outcomes in patients treated for a 12-month period.

**Material and Methods:** Thirty centers were enrolled from December 2018 to December 2019 within the open-label prospective EAP up to a maximum of 25 mg/kg per day. Adverse effects and liver function tests were assessed after 2 weeks; 1, 3, and 6 months of treatment; and periodically thereafter. Seizure endpoints were the percentage of patients with ≥50 and 100% reduction in seizures compared to baseline.

**Results:** A total of 93 patients were enrolled and included in the safety analysis. Eighty-two patients [27 (32.9%) DS, 55 (67.1%) LGS] with at least 3 months of treatment have been included in the effectiveness analysis; median previously failed antiseizure medications was eight. Pediatric and adult patients were uniformly represented in the cohort. At 3-month follow-up, compared to the 28-day baseline period, the percentage of patients with at least a 50% reduction in seizure frequency was 40.2% (plus 1.2% seizure-free). Retention rate was similar according to diagnosis, while we found an increased number of patients remaining under treatment in the adult group. CBD was mostly coadministered with valproic acid (62.2%) and clobazam (41.5%). In the safety dataset, 29 (31.2%) dropped out: reasons were lack of efficacy [16 (17.2%)] and adverse events (AEs) [12 (12.9%)], and one met withdrawal criteria (1.1%). Most reported AEs were somnolence (22.6%) and diarrhea (11.9%), followed by transaminase elevation and loss of appetite.

**Conclusions:** CBD is associated with improved seizure control also in a considerable proportion of highly refractory patients with DS and LGS independently from clobazam use. Overall, CBD safety and effectiveness are not dose-related in this cohort.

## Introduction

Cannabidiol (CBD) is a non-psychoactive phytocannabinoid derived from the *Cannabis sativa* plant with antiseizure effects through a still partially unknown mechanism that does not activate or bind directly cannabinoid receptors, unlikely to tetrahydrocannabidiol (1). Several mechanisms have been proposed to mediate antiseizure proprieties so far, including the inhibition of the GPR55 orphan receptor and adenosine reuptake, as well as the activation/desensitization of TRPV1 (2, 3). A pharmaceutical formulation of highly purified CBD has been recently approved by US Food and Drug Administration (FDA) (4) and European Medicine Agency (EMA) (5) for the treatment of seizures associated with two treatment-resistant epilepsies (TREs), Dravet (DS), and Lennox–Gastaut (LGS) syndromes, typically refractory to currently available antiseizure medications (ASMs) and more recently for the treatment of seizures associated with tuberous sclerosis (6). Pharmacokinetic and pharmacodynamic drug–drug interactions can occur between CBD and clobazam (CLB), with an up to 5-fold increase in *N*-desmethylclobazam plasma concentration. Notably, and in line with this observation, EMA authorization imposes the coadministration with CLB as a prescription rule in contrast to FDA. Subsequently, a meta-analysis indicated the lack of difference in seizure outcome in CLB-off patients (7); undoubtedly, any regulatory discrepancy should be addressed following convincing clinically relevant results.

CBD has demonstrated efficacy and an acceptable safety profile both in four phase III clinical trials and in expanded access programs (EAPs), also referred to as Compassionate Use Programs. Although, biased by the lack of a control group and open-label design, EAPs have the advantage to be more reflective of clinical practice and to facilitate access to innovative treatments before approval. We report the *interim* results on CBD safety and seizure outcomes from an Italian EAP.

## Materials and Methods

### Patient Population and Study Design

Thirty Italian epilepsy centers enrolled LGS and DS patients from December 2018 through an open-label prospective and ongoing EAP with eligibility criteria (Supplementary Material) comparable to placebo-controlled trials and other EAPs (8–10), with dosages up to a maximum of 25 mg/kg per day. The protocol was approved by each site (DM 07/09/2017; Italian Official Gazette on November 2, 2017), and written informed consent has been provided by patients or parents/caregivers. The study was conducted following the Good Clinical Practice guidelines and local standard operating procedures. Overall data collection has been approved by the Ethics Committee, Catanzaro, Italy, protocol no. 115/19.

### Procedures

Data were collected on all seizure types and according to the previous studies (8, 10, 11), convulsive seizures were defined as tonic, clonic, tonic–clonic, atonic, or secondary generalized. Non-convulsive seizures were defined as myoclonic, absence, or myoclonic–absence seizures, and focal seizures with or without impaired consciousness.

During a 4-week baseline period, diaries of all countable seizures have been provided by patients or parents/caregivers. Afterward, patients received an oral solution of purified CBD (100 mg/ml; Epidyolex GW Research Ltd.), starting dosage between 2 and 5 mg/kg per day up to 18–25 mg/kg per day, depending on the site.

Concomitant ASMs were recorded at baseline and during the treatment period. CBD and ASM dose modifications, as well as adding/removing co-ASMs, were allowed as clinically appropriated.

Visits have been performed after 2 weeks; 1, 3, and 6 months of treatment; and periodically thereafter. However, scheduled visits to assess treatment were programmed at 3, 6, 9, at 12 months.

Assessment of adverse events (AEs) and clinical laboratory parameters was performed approximately after 2 weeks; 1, 3 and 6 months of treatment; and periodically thereafter. AEs were classified using the Medical Dictionary for Regulatory Activities (MedDRA, version 22.0). All AEs have been reported and detailed as severe or leading to discontinuation as appropriate. Finally, the incidence of AEs has been reported according to concomitant ASMs.

### Assessment of Effectiveness

Seizure frequency has been provided per week since the previous visit, and efficacy outcomes were assessed at 3, 6, 9, and 12 months. According to other similar published studies (8, 11, 12), weekly seizure frequency was converted to frequency per 28 days (weekly frequency × 4). Percentage change in seizure frequency for each patient was calculated as ([seizure frequency per 28 days]–[seizure frequency at baseline])/[seizure frequency at baseline] × 100. Median percentage changes in seizure frequency were calculated due to interpatient variability (8, 11, 12).

Seizure endpoints were the percentage of patients with ≥50 and 100% reduction in monthly convulsive and total seizures compared to 4-week baseline (response rate). Additional variables assessed were episodes of status epilepticus, use of rescue medications, and hospital admissions.

Some sites assessed changes in electroencephalography before and during treatment. Furthermore, questionnaires on quality of life (i.e., QOLIE-31), sleep disturbance (i.e., Sleep Disturbance Scale for Children, Epworth Sleepiness Scale), behavior (Neurological Disorders Depression Inventory for Epilepsy, Child Behavior Check List, Beck Depression Inventory for Primary Care), and the Clinical Global Impression Scale have been collected. However, data have not been provided consistently through sites and have not been reported in the current analysis.

### Analysis

The sample size was based on patients' enrolment on each study site and not precalculated. Patients treated with at least one dose of CBD and post baseline evaluation have been assessed in the safety analysis. Effectiveness analysis was composed of all patients with at least 3 months of treatment. Kaplan–Meier curves have been built to evaluate CBD retention rates in effectiveness population and in patients with at least 1-month follow-up. The Mann–Whitney *U*-test for continuous variables and the two-tailed Pearson χ^2^ test or the Fisher test for categorical variables have been applied as appropriate. Finally, univariate and multivariate logistic regression analyses were carried out [odds ratios (ORs) and 95% confidence intervals (CIs)] to explore the variables independently associated with responder status at 3 and 12 months; variables included in the equation variables were significant in previous analysis or had a clinical interest. A *p* < 0.05 was considered significant for all variables. All the data were analyzed using SPSS software version 26.0 (SPSS Statistics; IBM Corp., Armonk, NY, USA).

## Results

### Clinical Features

**Figure 1:**
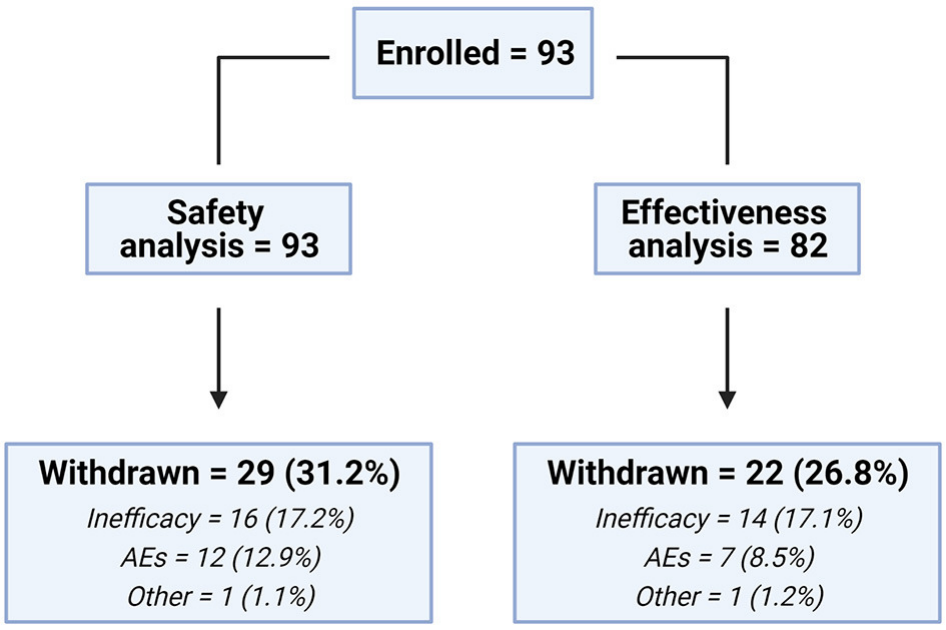
Patients' distribution flowchart. AEs, adverse events. Created with Biorender.com.

A total of 93 patients were enrolled in the EAP; the median number of patients per site was 3 (range = 1–11), and all patients have been included in the safety analysis. Eighty-two patients [27 (32.9%) DS, 55 (67.1%) LGS] with at least 3 months of treatment have been included in the effectiveness analysis. In the safety dataset, 29 (31.2%) dropped out; reasons were lack of efficacy [16 (17.2%)] and AEs [12 (12.9%)], and one met withdrawal criteria (1.1%; concomitant use of other cannabis-derived products) (Figure 1).

Overall, the mean (SD) treatment duration was 8.7 (4.1) months, and effectiveness data for the 12-month follow-up were available for 51 of 82 patients (62.2%). In both analysis groups, the mean age was 21 years (range = 3–56 years), about 32.0% had DS, and adults were 50.5% and the 52.4% in safety and effectiveness analyses, respectively. Demographic and clinical features at baseline are presented in Table 1. Patients' stratifications by diagnosis and age are detailed in Supplementary Tables 1, 2.

**Table 1:** Patients baseline demographic and clinical features.

	**Safety** ** (*n* = 93)**	**Effectiveness** ** (*n* = 82)**
Age (years), mean ± SD	21.4 ± 13.5	21.0 ± 13.1
Sex, male/female, *n* (%)	49 (52.7)/44 (47.3)	46 (56.1)/36 (43.9)
Body weight (kg), mean ± SD	50.8 ± 23.1	50.8 ± 21.9
Pediatrics/adults, *n* (%)	46 (49.5)/47 (50.5)	39 (47.6)/43 (52.4)
Diagnosis		
Dravet, *n* (%)	30 (32.3)	27 (32.9)
Lennox–Gastaut, *n* (%)	63 (67.7)	55 (67.1)
Concomitant ASMs taken at baseline, median (Q1–Q3)	3 (3–4)	3 (3–4)
Convulsive seizures/28 d, median (Q1–Q3)^*^	—	49 (12–147)
Total seizures/28 d, median (Q1–Q3)^*^	—	71.5 (23.6–181)

*ASMs, antiseizure medications*.

* *During 4-week baseline period*.

At baseline, a median of eight ASMs utilized before CBD administration has been reported, with the median number of concomitant ASMs at the time of CBD administration being 3 (range = 1–5).

Concomitant ASMs are detailed in Supplementary Table 3. The most common concomitant ASMs were valproic acid (62.2%, including sodium valproate), CLB (41.5%), lamotrigine (25.6%), and stiripentol (19.5%). The mean doses administered before CBD treatment were 19 (9.8) mg/day for CLB and 916 (557.7) mg/day for valproate.

### Seizure Outcomes

At baseline, the median (Q1, Q3) monthly frequency of convulsive and total seizures was 49 (12, 147) and 71.5 (23.6, 181) (Table 1). At the first 3-month follow-up, 24 patients (40.2%), compared to the 28-day baseline period, reported at least a 50% reduction in total-seizure frequency plus *one* patient seizure-free (1.2%).

**Figure 2:**
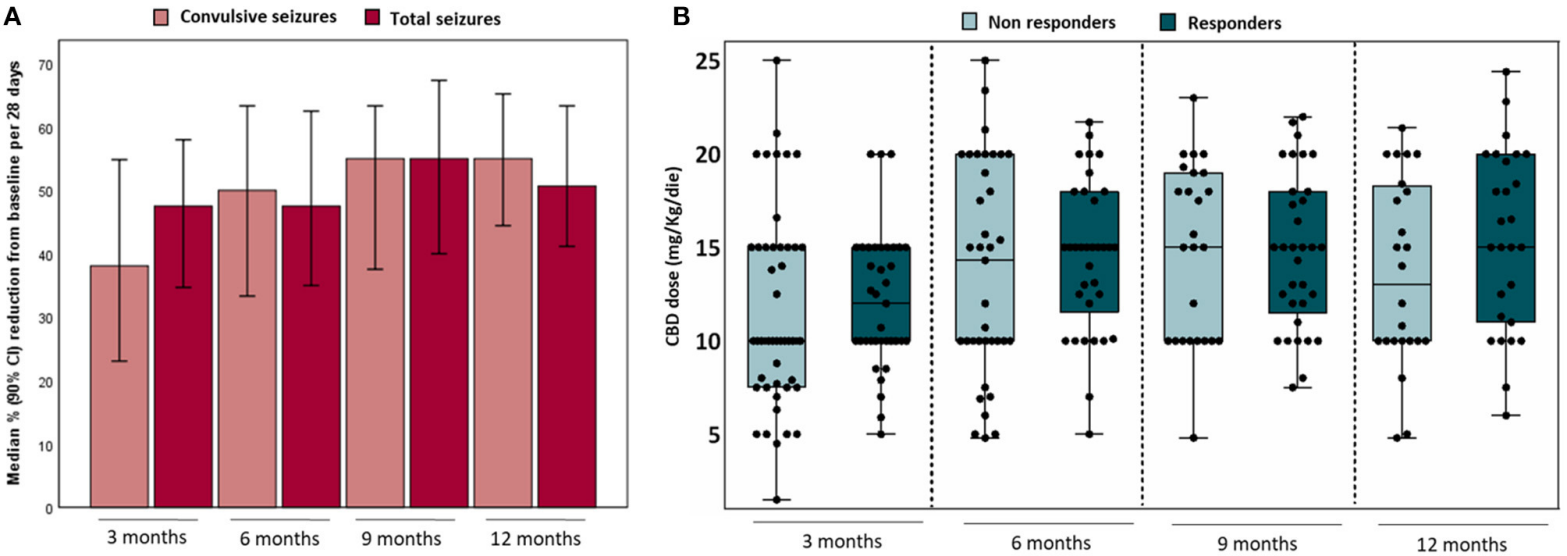
Percentage reduction in median seizures per 28 days from baseline in convulsive and total^#^ seizures for effectiveness analysis **(A)** and CBD doses related to achieving responder status at different outcomes **(B)**. ^#^Total seizures included convulsive seizures (i.e., clonic, tonic, tonic–clonic, atonic, focal secondary generalized) and non-convulsive seizures (i.e., myoclonic, absence, myoclonic-absence, focal with and without impaired consciousness). NR, non-responders; R, responders (≥50% frequency reduction and seizure-free).

At 12-month follow-up (51/82 patients, 62.2%), the percentage of patients with at least a 50% reduction in total-seizure frequency was 49.0% (plus 3.9% seizure-free), whereas 21.6% had a reduction <50%, 15.7% had no change, and 9.8% seizures worsening (Table 2). Median reductions of 50.7 and 55.0% in total and convulsive seizures frequencies have been reported (Figure 2A). No differences were highlighted in achieving responder status at 12 months in patients cotreated with CLB (*p* = 0.64) (Supplementary Figure 1).

**Table 2:** Treatment response rate for convulsive seizures (A) and total seizures (B).

	**Full cohort**	**Worsened**	**Unchanged**	** <50%**	**≥50%**	**Seizure-free**
**(A)**						
Outcome 3 months, *n* (%)	82 (100)	11 (13.4)	21 (25.6)	24 (29.3)	24 (29.3)	2 (2.4)
Outcome 6 months, *n* (%)	71 (86.5)	8 (11.3)	13 (18.3)	17 (23.9)	29 (40.8)	4 (5.6)
Outcome 9 months, *n* (%)	61 (74.4)	7 (11.5)	9 (14.7)	14 (22.9)	28 (45.9)	3 (4.9)
Outcome 12 months, *n* (%)	51 (62.2)	6 (11.7)	6 (11.7)	12 (23.5)	23 (45.1)	4 (7.8)
**(B)**						
Outcome 3 months, *n* (%)	82 (100)	10 (12.2)	18 (22.0)	20 (24.4)	33 (40.2)	1 (1.2)
Outcome 6 months, *n* (%)	72 (87.8)	6 (8.3)	14 (19.4)	17 (23.6)	32 (44.5)	3 (4.2)
Outcome 9 months, *n* (%)	61 (74.4)	3 (4.9)	10 (16.4)	13 (21.3)	33 (54.1)	2 (3.3)
Outcome 12 months, *n* (%)	51 (62.2)	5 (9.8)	8 (15.7)	11 (21.6)	25 (49.0)	2 (3.9)

*Total seizures included convulsive seizures (i.e., clonic, tonic, tonic–clonic, atonic, focal secondary generalized) and non-convulsive seizures (i.e., myoclonic, absence, myoclonic absence, focal with and without impaired consciousness). All response rate percentages are reported considering the total number of patients per follow-up. Seizure-free is not included in ≥50% cohort*.

The median dose of CBD between 3 and 12 months was 14 mg/kg per day. The CBD doses related to achieving responder status (defined as reduction ≥50% in seizure frequency plus seizure-free) at different follow-up are reported in Figure 2B; no difference was observed between responders and non-responders. Twenty patients (20/82; 24.4%) reduced the CBD dose at any time during follow-up. Approximately 25% of the patients taking concomitant CLB and/or valproate modified their dose from baseline during the study (Table 3).

**Table 3:** Dosing information coadministered ASMs.

**ASMs dose adjustment at all visits, *n* (%)**	**Valproate** ** (*n* = 51)**	**Clobazam** ** (*n* = 34)**	**Lamotrigine** ** (*n* = 21)**
Baseline dose stable	39 (74.5)	26 (76.5)	16 (76.2)
Baseline dose increased	1 (1.9)	0	0
Baseline dose decreased	8 (15.6)	1 (2.9)	3 (14.3)
Baseline dose increased and decreased	3 (5.9)	7 (20.6)	2 (9.5)

*ASMs, antiseizure medications*.

Univariate logistic regression was performed to determine the effects of several variables on achieving responder status at 3 and 12 months of treatment (Table 4). Multivariate logistic regressions using the variables included in the univariate analysis were performed. Both models explained 30% (Nagelkerke *R*^2^) of the variance to achieve responder status at 3 and 12 months. Only CLB use was independently associated with higher responder rate (OR = 4.04, CI = 1.1–14.5, *p* = 0.03) at 3 months but not at 12 months (Table 5). No variables have been significantly associated at 12 months.

**Table 4:** Univariate regressions with selected variables for clinical response.

	**Clinical response at 3 months**			**Clinical response at 12 months**		
	**OR**	**95% CI**	***p*-value**	**OR**	**95% CI**	***p*-value**
Age	1.02	0.98–1.05	0.21	0.99	0.95–1.04	0.90
Sex, female	2.85	1.15–7.09	**0.02**	1.85	0.59–5.78	0.28
Diagnosis (Lennox–Gastaut)	1.05	0.41–2.66	0.93	1.42	0.45–4.46	0.54
Pediatrics	0.52	0.21–1.28	0.15	0.83	0.26–2.63	0.75
Patients experienced AEs	1.55	0.64–3.77	0.33	0.77	0.25–2.33	0.64
CBD dose (3 or 12 months)	1.04	0.94–1.15	0.41	1.08	0.96–1.21	0.18
Concomitant ASMs	0.89	0.55–1.47	0.66	0.88	0.47–1.64	0.69
Cotreatment with clobazam	1.82	0.74–4.46	0.19	1.30	0.43–3.93	0.64
Cotreatment with stiripentol	0.40	0.12–1.37	0.14	0.68	0.18–2.60	0.57
Cotreatment with valproate	0.63	0.25–1.56	0.32	0.47	0.12–1.37	0.15
Cotreatment with lamotrigine	1.40	0.51–3.80	0.50	1.26	0.36–4.36	0.71
Convulsive seizures frequency at baseline	0.99	0.99–1.00	0.19	1.00	0.99–1.01	0.17
Total seizures frequency at baseline	1.00	0.99–1.00	0.57	1.00	0.99–1.00	0.09

*ASMs, antiseizure medications; CBD, cannabidiol; AEs, adverse events. Bold values are statistically significant*.

**Table 5:** Multivariate regressions with selected variables for clinical response.

	**Clinical response at 3 months**			**Clinical response at 12 months**		
	**OR**	**95% CI**	***p*-value**	**OR**	**95% CI**	***p*-value**
Age	1.01	0.94–1.08	0.86	0.99	0.19–1.07	0.89
Sex, female	2.30	0.76–6.91	0.14	1.66	0.38–7.23	0.50
Diagnosis (Lennox–Gastaut)	0.41	0.08–2.07	0.28	0.28	0.04–2.24	0.23
Pediatrics	0.73	0.14–3.81	0.71	0.41	0.05–3.26	0.40
Patients experienced AEs	0.97	0.30–3.17	0.97	1.12	0.23–5.24	0.88
CBD dose (3 or 12 months)	1.06	0.93–1.21	0.34	1.14	0.98–1.31	0.08
Concomitant ASMs	0.74	0.38–1.40	0.35	1.01	0.43–2.36	0.98
Cotreatment with clobazam	4.04	1.12–14.57	**0.03**	3.39	0.58–19.87	0.17
Cotreatment with stiripentol	0.23	0.04–1.37	0.11	0.69	0.08–5.65	0.73
Cotreatment with valproate	0.62	0.18–2.08	0.44	0.23	0.04–1.24	0.08
Cotreatment with lamotrigine	2.80	0.72–10.7	0.14	3.98	0.65–24.45	0.13
Convulsive seizures frequency at baseline	0.96	0.92–1.01	0.10	1.03	0.98–1.08	0.21
Total seizures frequency at baseline	1.02	0.99–1.06	0.14	1.01	0.98–1.03	0.58

*Nagelkerke R^2^ = 0.31 for 3 months and 0.30 for 12 months. All the variables have been included in the model. ASMs, antiseizure medications; CBD, cannabidiol; AEs, adverse events. Bold values are statistically significant*.

### CBD Retention

**Figure 3:**
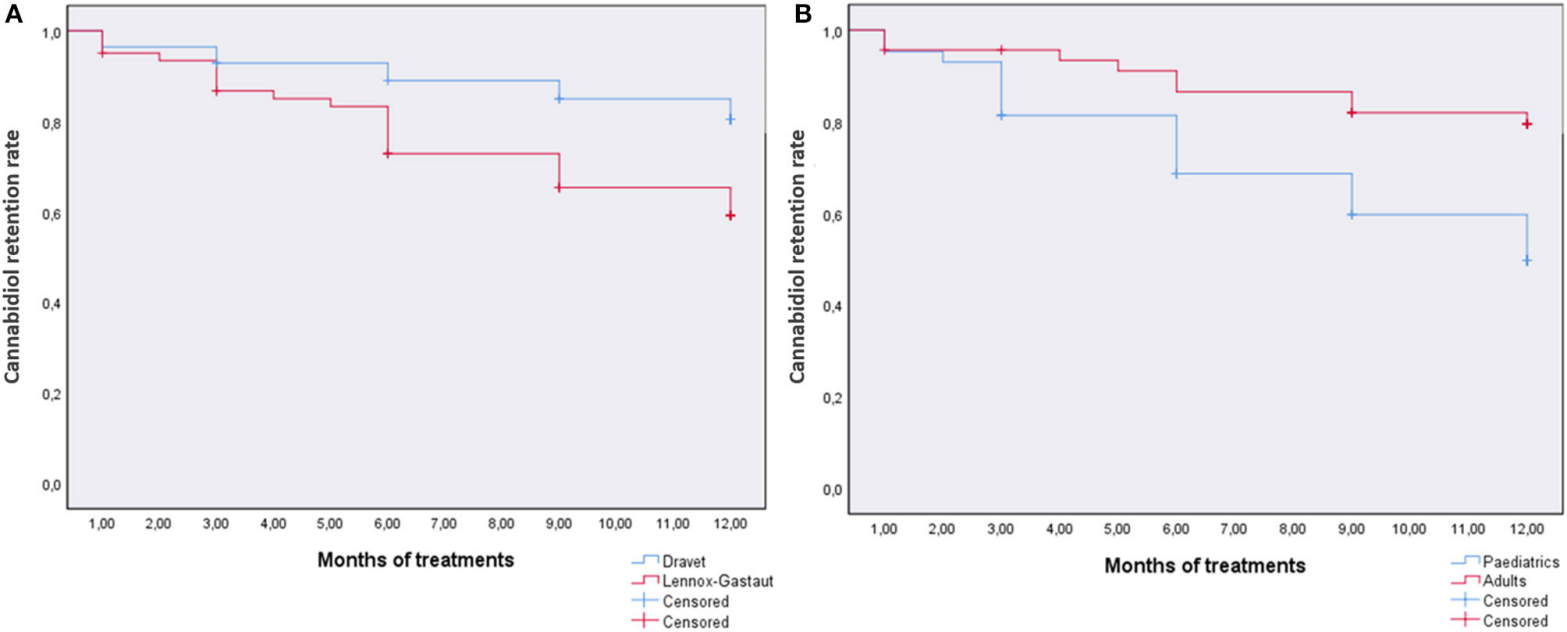
The retention rate of cannabidiol in patients with at least 1-month follow-up stratified by diagnosis (Dravet syndrome and Lennox–Gastaut syndrome) **(A)** or age (pediatrics and adults) **(B)**.

In patients with at least 1 month of treatment, the overall retention rate was 68.5%, and log-rank tests were run to determine differences in the CBD retention rate for diagnosis (DS and LGS) or age (pediatrics and adults). The survival distribution was statistically significantly different for age, χ^2^ = 7.38, *p* = 0.007 (80.4% retention rate for patients ≥18 years), whereas no statistical significance was reached for diagnosis χ^2^ = 3.04, *p* = 0.06 (82.1% retention rate for DS) (Figure 3). Notably, when considering the diagnosis in the age subgroups, DS pediatric patients have a higher retention rate than LGS patients (χ^2^ = 9.96, *p* = 0.002), whereas no difference was observed in adult patients (χ^2^ = 0.03, *p* = 0.87) (Supplementary Figure 2).

### Tolerability

In the safety analysis, 48 patients (51.6%) experienced at least one AE. Overall, the most common AEs reported were somnolence [21 (22.6%)] and diarrhea [11 (11.8%)], followed by elevated liver enzymes (alanine aminotransferase/aspartate aminotransferase >3 upper than the normal limit) (10, 10.7%) and loss of appetite (8, 8.6%) (Table 6). Eight AEs (8.6%) have been classified as serious, with the most common being status epilepticus (9.6%) and vomiting (2.1%); 12 AEs [12/91 (13.2%)] led to CBD discontinuation. AEs are detailed in Supplementary Tables 4, 5. Patients with elevated liver enzymes or hyperammonemia [occurred in 10 (10.7%) and 7 patients (7.7%), respectively] were always cotreated with valproate. Somnolence occurred in 27.5% of patients taking CLB (11/40) compared to 15.1% (8/53) not cotreated. No thrombocytopenia (i.e., platelets count <140,000/μL) has been reported.

**Table 6:** Summary of adverse events in safety analysis.

	**CBD dose (mg/kg per day)**			
	**0–10** ** (*n* = 28)**	**11–15** ** (*n* = 29)**	**16–25** ** (*n* = 36)**	**All** ** (*n* = 93)**
Overall AE rate, *n* (%)	25 (89.3)	19 (65.5)	4 (11.1)	48 (51.6)
Overall serious AE rate, *n* (%)	3 (10.7)	4 (10.3)	1 (2.7)	8 (8.6)
AEs leading to CBD discontinuation, *n* (%)	4 (14.3)	6 (20.6)	2 (5.5)	12 (12.8)
**AEs reported** **≥2% in any group**				
Somnolence, *n* (%)	12 (42.8)	7 (24.1)	2 (5.5)	21 (22.6)
Diarrhea, *n* (%)	3 (10.7)	3 (10.3)	5 (13.8)	11 (11.8)
Transaminases elevated, *n* (%)	4 (14.3)	3 (10.3)	3 (8.3)	10 (10.7)
Status epilepticus, *n* (%)	1 (3.5)	5 (17.2)	3 (8.3)	9 (9.6)
Loss of appetite, *n* (%)	6 (21.4)	1 (3.4)	1 (2.7)	8 (8.6)
Hyperammonemia, *n* (%)	5 (17.8)	1 (3.4)	1 (2.7)	7 (7.5)
Balance disorder, *n* (%)	3 (10.7)	2 (6.8)	1 (2.7)	6 (6.4)
Irritability, *n* (%)	0	3 (10.3)	1 (2.7)	4 (4.3)
Vomit, *n* (%)	2 (7.1)	0	1 (2.7)	3 (3.2)

## Discussion

In our cohort of highly treatment-resistant patients with DS and LGS, add-on treatment of CBD for 12 months was associated with a reduction in seizure frequency and was generally well-tolerated.

Overall, the percentage of patients achieving a seizure reduction ≥50% for total seizures comprised between 41.4% (34/82 patients) at 3 months and 52.9% (27/51 patients) at 12 months. Our results are in line with the 38–52% reported in several studies involving different TREs (8, 12) and the 43–50% in an EAP with DS and Lennox syndrome only (10). Furthermore, a consistent percentage of patients achieved a seizure-free status compared to baseline after 3 months of treatment and during the 12-month follow-up period.

No differences have been highlighted in median seizure frequency reductions comparing patients on CLB and those without, as well as in responder status achievement. However, CLB use has been associated with higher responder status (only at 3 months), as already reported (12). These findings confirm that CBD has antiseizure activity independent of concomitant CLB, but it is unknown to which extent CBD efficacy is enhanced (13).

The AE rates were lower (51.6%) than those reported in other EAPs and randomized clinical trials (79–94%), although, the most common reported AEs, somnolence, and diarrhea, were in line with the literature. On the other hand, an unexpected higher percentage of patients discontinued CBD because of AEs (12.8%), considering reported rates of 5.1, 8, and 3% in previous EAPs. However, one-third of discontinuations due to AEs belong to a single enrolling site, and this might overestimate the overall rate. Withdrawals for any reason were distributed regularly through the study follow-up period. The most common serious AEs reported, status epilepticus (9%) and vomiting (2%), were consistent with previous studies and randomized controlled trials (RCTs) (2, 14).

Notably, the overall incidence of AEs was higher in the group administered <10 mg/kg per day than the other dose group, in sharp contrast to the suggested dose effect (mainly for somnolence) reported in previous studies. Recently, one study has reported thrombocytopenia in one-third of patients treated concurrently with CBD and valproic acid (15). In our study, no cases of thrombocytopenia occurred, even though 62% of patients were cotreated with CBD and VPA.

Retention rate is generally used as a combined measure of effectiveness, tolerability, and patient/clinician preference. During the follow-up period, 68.5% of the patients with at least 1 month of treatment remained on CBD, relatively in line with the other EAPs at 12 months (~60%) (11) and studies with no TREs (63–81%) (16). Bearing in mind the limitation of the low number of patients treated, we found that the retention rate for adults was significantly higher than that for pediatric patients; this parameter is not accompanied by a significant difference on seizures, but it is worth noting that in our ancillary study on CBD plasma concentrations, we observed that concentration/dose ratio is significantly lower in patients younger than 18 years (17). Also in this latter case, no correlation was found between dose, plasma concentration, and efficacy, and further studies are warranted to understand whether this low trough CBD concentration is meaningfully linked to efficacy and safety.

The median CBD dose (14 mg/kg per day) remains stable at all follow-ups, although, 20 patients reduced the dose as allowed by the protocol. Unfortunately, the reason for reductions was not consistently reported by sites and could not be analyzed.

CBD has well-known bidirectional drug–drug interactions with CLB (increasing nordesmethylclobazam and 7-hydroxy-CBD) and valproate (probably pharmacodynamic rather than pharmacokinetic interactions) (18, 19), and several AEs have been reported due to drug–drug interactions. In our cohort, all the patients reporting transaminase elevation or hyperammonemia were taking concomitant valproate, further confirming the role of this interaction in the development of such AEs, as reported in the aforementioned EAPs and RCTs. As expected, somnolence has been experienced twice in patients on concomitant CLB compared to patients without, but no patients withdrew due to somnolence.

Main limitations of this study are open-label design and uncontrolled EAP. Furthermore, reporting methods could be different among enrolling sites and motivation for CBD, or concomitant ASM dose reductions were not consistently reported. However, EAP can provide useful data being closer to clinical practice compared to randomized clinical trials and therefore more generalizable.

Of note, we found a high rate of patients on treatment at 12 months without a clear improvement in seizure count, raising the question whether other aspects and effects of CBD may have a positive impact on the overall clinical state. An alternative explanation is that Italian doctors, in the context of an EAP and of a public health–based medical system where no restrictions exist in the duration of a treatment irrespective of its cost and actual efficacy, have a careless attitude toward withdrawing it, even after it has proven ineffective. On the other hand, public interest and expectancy in cannabis-based/derived therapies have been rising in the past 10 years and may have influenced patients and caregivers in a similar manner (20).

In conclusion, we confirm CBD effectiveness and tolerability in highly refractory DS and LGS patients also without the concomitant use of CLB. Of note, dose dependency for both efficacy and tolerability is not evidenced by our data. Finally, whether other potential CBD effects on the central nervous system (e.g., anxiolytic, antipsychotic) (21) may have a role in clinical practice warrants further research, and other parameters than seizure outcome may be worth a clinical evaluation (22, 23).

## Data Availability Statement

The original contributions presented in the study are included in the article/Supplementary Material, further inquiries can be directed to the corresponding author/s.

## Ethics Statement

The protocol was approved by each site (DM 07/09/2017; Italian Official Gazette on November 2nd, 2017) and written informed consent has been provided by patients or parents/caregivers. The study was conducted following the Good Clinical Practice guidelines and local standard operating procedures. Overall data collection has been approved by the Ethics Committee, Catanzaro, Italy, protocol number 115/19.

## Author Contributions

ER and OM contributed to conception and design of the study. LI and GA organized the database. LI performed the statistical analysis. ER, LI, and PS wrote the manuscript. DB, FB, PB, AB, MPC, GCa, EC, MC, AC, DC, GCr, VD, MFD, MD, Gd'O, ME, CG, AM, TG, RG, ML, AL, FM, SM, RM, MN, NPil, DP, FR, AR, MS, AS, NPie, PS, ET, LT, AP, CZ, and OM provide patients' data. All authors contributed to manuscript revision, read, and approved the submitted version.

## CBD LICE Italy Study Group

Clementina Boniver (Paediatric Neurology and Neurophysiology Unit, Department of Women's and Children's Health, University Hospital of Padua, Padua, Italy), Maria Bottitta (Oasi Research Institute IRCCS, Troina, Italy), Carlo Di Bonaventura (Neurology Unit, Department of Human Neurosciences, “Sapienza” University, Rome, Italy), Viola Doccini (Pediatric Neurology, Neurogenetics, and Neurobiology Unit and Laboratories, A. Meyer Children's Hospital, Florence, Italy), Roberta Epifanio (Clinical Neurophysiology Unit, IRCCS Eugenio Medea, Scientific Institute, Lecco, Italy), Giovanni Falcicchio (Department of Basic Medical Sciences, Neurosciences and Sense Organs, University of Bari, Bari, Italy), Massimo Mastrangelo (Department of Pediatric Neurology, V. Buzzi Children's Hospital, Milan, Italy), Sara Matricardi (Child Neurology and Psychiatry Unit, G. Salesi Children's Hospital-University of Ancona, Ancona, Italy), Ludovica Pasca (Department of Child Neurology and Psychiatry, IRCCS Mondino Foundation, Pavia Italy), Federica Pondrelli (Department of Biomedical and Neuromotor Sciences, University of Bologna, Bologna, Italy), Patrizia Pulitano (Department of Human Neurosciences, Sapienza University, Rome, Italy), Antonella Riva (Pediatric Neurology and Muscular Diseases Unit, IRCCS ‘G. Gaslini' Institute, Genoa, Italy; Department of Neurosciences, Rehabilitation, Ophthalmology, Genetics, Maternal, and Child Health, University of Genova, Genova, Italy), Paola Russo (Department of Neuroscience, Reproductive, and Odontostomatological Sciences, Epilepsy Centre, University of Naples Federico II, Naples, Italy), Nicola Specchio (Department of Pediatric Neuroscience, Fondazione IRCCS Istituto Neurologico Carlo Besta, Milano, Italy), Silvia Spolverato (Child Neuropsychiatry, Department of Surgical Sciences, Dentistry, Gynecology, and Pediatrics, University of Verona, Verona, Italy), Aglaia Vignoli (Department of Health Sciences, Epilepsy Center, San Paolo Hospital, University of Milan, Milan, Italy), Maurizio Viri (Child Neuropsychiatry Department, Maggiore della Carità University Hospital, Novara, Italy), Lilia Volpi (IRCCS Istituto delle Scienze Neurologiche di Bologna, Unit of Neurology, Bellaria Hospital, Bologna, Italy).

## Conflict of Interest

FB has participated in clinical trials for GW Pharmaceuticals; received research grants, speaker fees or participated to advisory boards for Eisai, Cyberonics, UCB Pharma and Bial. MPC Canevini has participated advisory boards and/or received research fundings from UCB Pharma, Eisai, Italfarmaco, Cyberonics, Novartis, and the European Union. AC has received speaker fees by Eisai. CB has received speaker fees from Eisai, UCB Pharma, FB Health and Sandoz. Gd'O has served on the advisory board of Eisai. CG has received research grants and/or speaker fees from UCB Pharma, Eisai and Bial. TG received a speaker fee from GW Pharmaceuticals. RG has received consulting fees and speaker honoraria from Zogenix, Biomarin, UCB, Eisai, Novartis, GW Pharma, and Biocodex. OM has received consulting fees and speaker honoraria by Bial, Eisai, GW Pharmaceuticals and UCB Pharma. AL has received speaker's or consultancy fees from Eisai, Mylan, Sanofi, Bial, GW Pharmaceuticals and UCB Pharma. PP received speaker's fees from Eisai and UCB Pharma. AR received consulting fees from GW Pharmaceuticals. ER has received speaker fees and/or fundings and has participated in advisory boards for Eisai, Pfizer, GW Pharmaceuticals, UCB Pharma, Arvelle Therapeutics. NS has received grant support and fees for advisory board participation from GW Pharmaceuticals. PS developed within the framework of the DINOGMI Department of Excellence of MIUR 2018-2022 (legge 232 del 2016) and received speaker fees and participated at advisory boards for Biomarin, Zogenyx, GW Pharmaceuticals, Neuraxpharma; he also received research funding by ENECTA BV, GW Pharmaceuticals, Kolfarma srl., Eisai. ET has received speaker's fees from Eisai and Sandoz. AV received speaker's fees from Eisai, Italfarmaco, and GW Pharmaceuticals. MV received speaker's fees from Eisai and GW Pharmaceuticals. The remaining authors declare that the research was conducted in the absence of any commercial or financial relationships that could be construed as a potential conflict of interest.
