# Supplementary material for: Results From an Italian Expanded Access Program on Cannabidiol Treatment in Highly Refractory Dravet Syndrome and Lennox–Gastaut Syndrome
Source: Front Neurol. 2021 May 20;12:673135. doi: 10.3389/fneur.2021.673135 (PMC8173151; doi:10.3389/fneur.2021.673135)
Supplement: Supplementary file 1 [file Data_Sheet_1.pdf]

To access the expanded access program for cannabidiol, patients must fulfill the following criteria:

1. Aged 2 years and above;
2. Dravet syndrome or Lennox-Gastaut syndrome diagnosis;
3. Currently taking between 1 and 4 other ASMs, with a stable antiseizure treatment for the previous 4 weeks (including ketogenic diet and vagus nerve stimulation);
4. Lack of seizures control with 2 or more prior ASMs treatment failed
5. Written informed consent provided by the participant and/or parent(s)/caregiver(s).

Patients with any of the following conditions cannot access the expanded access program for cannabidiol:

1. Pregnancy, lactation, and risk of pregnancy
2. Clinically significant unstable medical or psychiatric conditions that may place patient's safety at risk;
3. Clinically significant liver disease or serum aminotransferase (ALT or AST) > 3 times the upper limit of the normal range or total bilirubin > 2 times the upper limit of the normal range or international normalized ratio (INR) > 1.5.
4. Known or suspected intolerance or hypersensitivity to cannabinoids or any of the excipients of the medicinal product such as sesame oil;
5. Stable felbamate dosing < 1 year
6. Current use (or use in the previous 2 months) of recreational or medicinal cannabis, or synthetic cannabinoid-based medications.
7. Alcohol abuse < 2 years before commencing cannabidiol initiation

Table S1. Diagnosis differences in safety and effectiveness population.

	**Safety population (93)**			**Effectiveness population (82)**		
	**Dravet**	**Lennox-Gastaut**	*p value*	**Dravet**	**Lennox-Gastaut**	*p value*
**Patients**, n (%)	30 (32.3)	63 (67.7)		27 (32.9)	55 (67.1)	
**Age** [years], mean ± SD	15.9 ± 9.5	23.5 ± 13.9	*0.02*	15.9 ± 9.6	24.1 ± 14.4	*0.02*
**Sex** M/F, n (%)	18 (60)/12 (40)	31 (49.2)/32 (50.8)	*0.33*	17 (63) /10 (37.0)	29 (52.7) / 26 (47.3)	*0.38*
**Paediatrics/adults,** n (%)	19 (63.3)/11 (36.7)	27 (42.9)/36 (57.1)	*0.65*	17 (63.0) /10 (37.0)	22 (40) / 33 (60)	*0.05*
**Concomitant-ASMs,** median (Q1-Q3)	3 (2-3)	3 (3-4)	*0.19*	3 (2-3)	3 (3-4)	*0.13*
**Patients experienced AEs**, n (%)	14 (29.2)	34 (70.8)	*0.51*	13 (48.1)	30 (54.5)	*0.59*
**Overall AEs**, median (Q1-Q3)	0 (0-1.2)	1 (0-2)	*0.39*	0 (0-1)	1 (0-2)	*0.33*
**Convulsive seizures/28d**, median (Q1- Q3) *	-	-		11.2 (4-36)	72 (40-168)	*0.0001*
**Total seizures/28d**, median (Q1-Q3) *	-	-		14 (4-42)	118 (57.2-224)	*0.0001*

ASMs, antiseizure medications; AEs, adverse events.

Table S2. Paediatrics and adults in safety and effectiveness analysis.

	Safety population (93)			Effectiveness population (82)		
	**Paediatrics**	**Adults**	*p value*	**Paediatrics**	**Adults**	*p value*
**Patients**, n (%)	46 (49.5)	47 (50.5)		39 (47.6)	43 (52.4)	
**Age** [years], mean ± SD	10.5 ± 4.1	31.3 ± 10.6	/	10.3 ± 4.1	31.5 ± 10.9	/
**Sex** M/F, n (%)	27 (58.7)/ 19 (41.3)	22 (46.8)/ 25 (53.2)	*0.25*	25 (61.1)/ 14 (35.9)	21 (48.8)/ 22 (51.2)	*0.16*
Diagnosis			*0.65*			*0.05*
**Dravet**, n (%)	19 (41.3)	11 (23.4)		17 (43.6)	10 (23.3)	
**Lennox-Gastaut**, n (%)	27 (58.7)	36 (76.6)		22 (56.4)	33 (76.6)	
**Concomitant-ASMs,** median (Q1-Q3)	3 (2-3)	3 (3-4)	*0.01*	3 (2-3)	3 (3-4)	*0.02*
**Patients experienced AEs**, n (%)	18 (37.5)	30 (62.5)	*0.17*	16 (37.2)	27 (62.8)	*0.05*
**Overall AEs**, median (Q1-Q3)	0 (0-1)	1 (0-2)	*0.003*	0 (0-1)	1 (0-2)	*0.01*
**Convulsive seizures/28d**, median (Q1-Q3) *	-	-		71 (11 -160)	44 (20-92)	*0.61*
**Total seizures/28d**, median (Q1-Q3) *	-	-		71 (12-192)	72 (40-168)	*0.43*

ASMs, antiseizure medications; AEs, treatment emergent adverse events.
**during 4-weeks baseline period*

Table S3. Effectiveness population - Co-administered ASMs ≥1%.

Specific ASM	n (%)
Valproate	51 (62.2)
Clobazam	34 (41.5)
Lamotrigine	21 (25.6)
Stiripentol	16 (19.5)
Phenobarbital	14 (17.1)
Topiramate	14 (17.1)
Clonazepam	11 (13.4)
Rufinamide	11 (13.4)
Carbamazepine	9 (11)
Ethosuximide	9 (11)
Lacosamide	9 (11)
Perampanel	8 (9.8)
Zonisamide	8 (9.8)
Levetiracetam	7 (8.5)
Nitrazepam	7 (8.5)
Felbamate	6 (7.3)
Brivaracetam	3 (3.7)
Diazepam	3 (3.7)
Eslicarbazepine	2 (2.4)
Oxcarbazepine	2 (2.4)
Phenytoin	1 (1.2)
Vigabatrin	1 (1.2)

ASMs, antiseizure medications.

Table S4. Safety population – Specific adverse events (AEs)

Specific AE	***n (%)***
Somnolence	21 (22.6)
Diarrhoea	11 (11.8)
Transaminases elevated	10 (10.7)
Status epilepticus	9 (9.6)
Loss of appetite	8 (8.6)
Hyperammonaemia	7 (7.5)
Balance disorder	6 (6.4)
Irritability	4 (4.3)
Vomit	3 (3.2)
Asthenia	2 (2.1)
Aggressivity	2 (2.1)
Nausea	2 (2.1)
Sialorrhea	2 (2.1)
Weight increase	1 (1.1)
Ataxia	1 (1.1)
Constipation	1 (1.1)
Cutaneous rush	1 (1.1)
Insomnia	1 (1.1)
Psychomotor agitation	1 (1.1)
Weight decrease	1 (1.1)
Slowdown	1 (1.1)
Other	5 (5.4)

Percentage on patients in safety analysis (93). Reported by Medical Dictionary for Regulatory Activities (MedDRA) preferred term.
AEs, adverse events.

Table S5. Safety population –Adverse events (AEs)

	**AEs (n = 48)**	***p* value**
**Age** [years], mean ± SD	24.9 ± 13.7	0.02
**Sex** M/F, n (%)	19 (38.8)/ 29 (65.9)	0.009
**Paediatrics/adults,** n (%)	18 (39.1)/ 30 (63.8)	0.02
Diagnosis		0.51
**Dravet**, n (%)	14 (46.7)	
**Lennox-Gastaut**, n (%)	34 (53.0)	
**Overall adverse events,** median (Q1-Q3**)**	2 (1-2)	/
**AEs leading discontinuation**, n (%)	12 (13.2)	/
**Concomitant ASMs**, median (Q1-Q3)	3 (3-4)	0.06
1	3 (6.3)	
2	5 (10.4)	
3	21 (43.8)	
4	14 (29.2)	
5	5 (10.4)	

ASMs, antiseizure medications; AEs, treatment emergent adverse events.

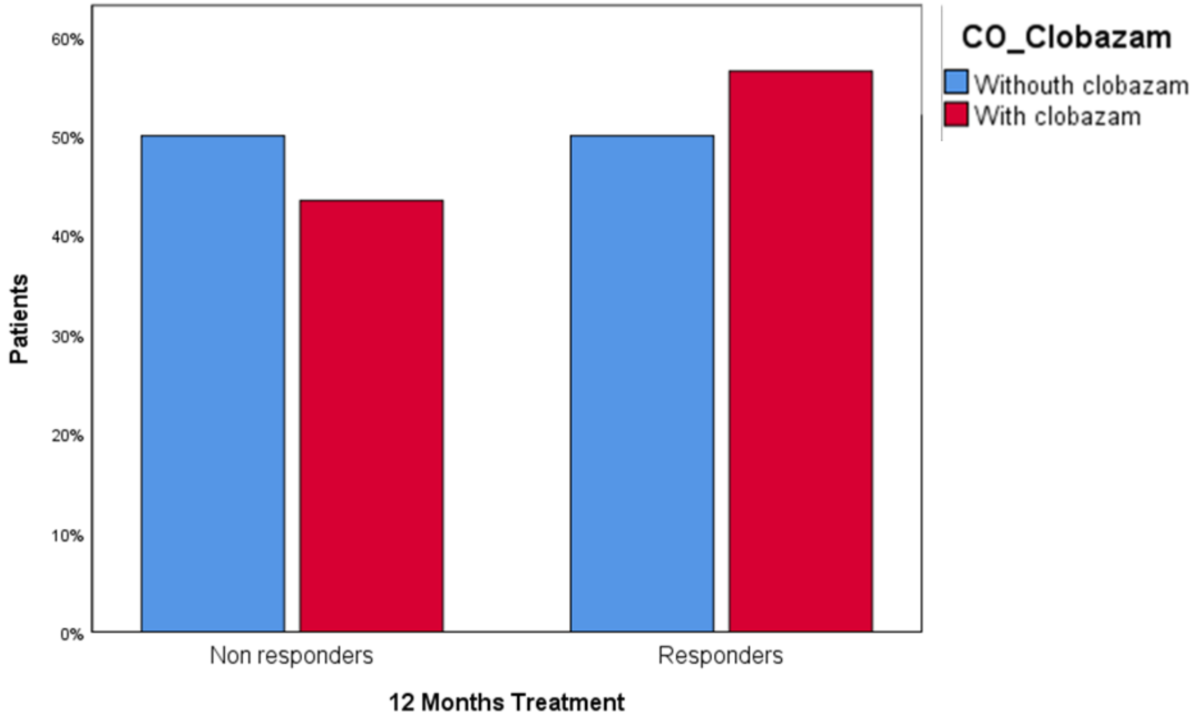

Figure S1. Responder status subdivided by clobazam use at 12 months follow-up.

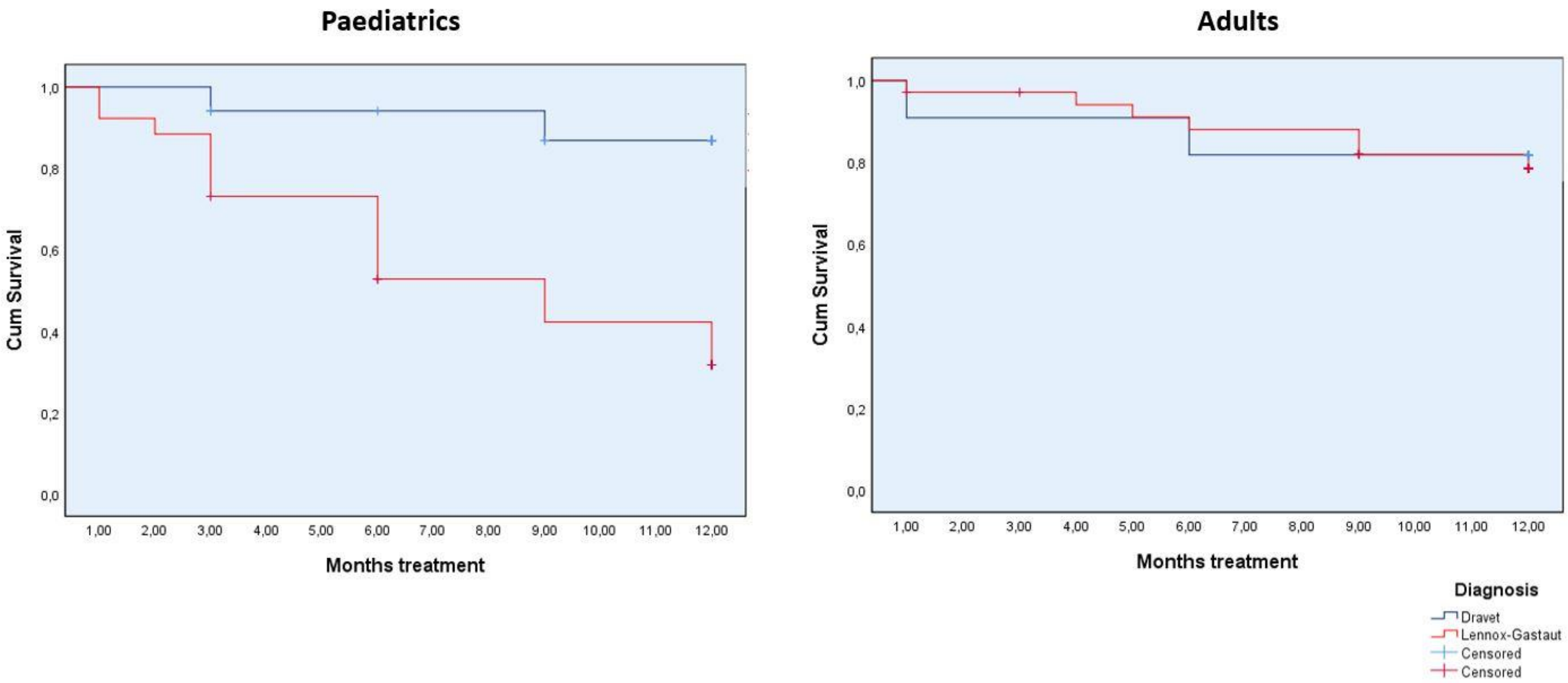

Figure S2. Retention rate of cannabidiol in patients with at least 1-month follow-up in age sub-population stratified by diagnosis.
